# Legislation for Midwives

**Published:** 1900-03-31

**Authors:** 


### The Institutional Workshop.

### LEGISLATION FOR MIDWIVES.

From a Correspondent.

(*Continued from page 420.*)

It was hardly to be expected that midwives should have improved in status under the course of events which deprived them of the most lucrative part of their business. Gradually, as all who could afford the higher charges of medical practitioners forsook the humbler offices of their own sex, midwives became less important personages, and as a natural consequence the craft fell into the hands of a lower and even less well instructed class than before. A counteracting tendency was, however, at work. About the middle of the eighteenth century a great panic arose, due to imperfect registration of births and deaths, that the population of England was steadily decreasing. To remedy this state of things, which was the more disastrous, had there been any truth in it, from the large number of men required at that time in the navy and for foreign service, a great effort was made to encourage the growth of population. From the middle of the century, within a period of fifty years, all the great lying-in hospitals were founded in quick succession, and the avowed object of these charities was not only to encourage large families by relieving mothers of all anxiety in their lying-in, but to lessen the loss of infant life by the training of midwives.

The chequered career of the lying-in hospitals need not here be recalled. It is true that for reasons only clearly understood within the last twenty years they produced among the class they desired to benefit a frightful mortality. But it is also true that they effected from the very first a valuable work for the nation by the systematic instruction of pupil midwives. On this neutral ground for the first time doctor and midwife were brought together in their natural relative position of teacher and pupil. The opportunity of extended clinical observation afforded by these institutions was readily grasped by the best doctors of the day, and under their instruction a succession of women were sent out for the service of the public, trained as completely as the knowledge of the day admitted. It is impossible to exaggerate the service which has been rendered by these voluntary charities in the education of midwives. Over a hundred years before trained nursing was developed in any other branch of practice, the great lying-in hospitals of London, Dublin, and other towns were turning out year after year bands of skilled women trained by actual residence under the eye of the surgeon in all that was then deemed essential for the safety of the mother and infant. No promoters of legislation for midwives can afford to disregard the fact that this widely-extended system of education, dating back from the year 1757, is still in force, and has never been interrupted, knowing no change beyond what is implied in a steady rise of standard in the teaching, and a very perceptible improvement in the class of candidates for instruction.

It will not be denied that a distinction is necessary to be observed between legislation for a body of well-trained workers, into whose ranks unworthy members have intruded, and legislation for a body of unskilled workers whom it is desired to improve. Although too many of the second order are to be found in their ranks, it is under the first aspect that midwives may fairly claim to be considered.

Were it otherwise, were all or nearly all, ignorant of their duties, it is to be feared legislation could do very little. Legislation may decree that licences should be granted, but it cannot call into being women qualified to receive the licence. Legislation may appoint examiners and draw up courses of instruction, but it cannot create well equipped schools, insure the co-operation of teachers capable of imparting the required information, nor attract to any calling the class of persons likely to do it credit. All this must be very slow work; it is the really essential part in the improvement of any profession, and in England at any rate, it will be found almost invariably to have been the outcome of voluntary as distinguished from State effort. Consequently, in such cases, should legislation be required, the first duty of its promoters is to respect the fabric already reared.

We are fond in this country of scourging ourselves with the belief that "they order this matter better in France" or elsewhere. It has been often urged that this is the only country in which midwives are uncontrolled by the State, and the argument places us at first sight in a very inferior position to other civilised governments. Yet there is good evidence to show that no country in Europe owns so large a number of women who have undergone special and exact training in this craft as England, and in no country is the standard of training higher.

If any proof were required that the mere issuing of licences and passing of examinations is ineffectual to raise the calling, it is only necessary to study the condition of affairs existing but a short time ago in two very much registered countries, France and Germany.

In France the registration of midwives dates as far back as 1803. They are ranged in two classes, and candidates for even the lowest order must attend two or three courses of instruction, must be examined by a "jury" in theory and practice, and must register their diplomas in their district under various pains and penalties. The whole profession has been placed under complete supervision and control, and every contingency is provided for in a thoroughly comprehensive manner. Yet what has been the result of this admirable paper organisation? The highest French authorities concur in pronouncing "the scientific standard of midwifery in the provinces and country towns is so low as to be practically non-existent; the greatest merit of the midwives is to know how to swaddle the baby."

Precisely the same result ensued in Germany, where, after many years of stringent legislation, the standard in country places was declared by Siebold to be beneath contempt, and the midwives to be utterly ignorant of their craft.

It is much to be feared that a mere system of registration would leave the midwives exactly where they were. The registered midwife would be no wiser than before she received her diploma. To meet the urgent needs of the poor a large body of these women must be allowed to practice. In remote country places where their ignorance may very probably be greatest, their services will be most imperatively required. An ill-judged measure might lead to the establishment of a very low standard of qualification, and unless it contained a provision for grants in aid of instructing the poorer class of widwives it would leave the case of the poorer mothers, about whom there is alone question, practically unrelieved.

